# Edmund John Crampin 1973–2021

**DOI:** 10.1007/s11538-021-00987-0

**Published:** 2022-01-29

**Authors:** Philip K. Maini, Peter J. Hunter, Peter J. Gawthrop, Nic P. Smith

**Affiliations:** 1 Wolfson Centre for Mathematical Biology, Mathematical Institute, University of Oxford, Oxford, OX2 6GG UK; 2 Auckland Bioengineering Institute, University of Auckland, Auckland, New Zealand; 3 Systems Biology Laboratory, Department of Biomedical Engineering, Faculty of Engineering and Information Technology, University of Melbourne, Victoria, 3010 Australia; 4 Systems Biology Laboratory, School of Mathematics and Statistics, University of Melbourne, Victoria, 3010 Australia; 5 Queensland University of Technology, Brisbane, Australia

Edmund Crampin was born in Bletchley in Buckinghamshire, close to the Open University where his father was a mathematician. He trained as a physicist at Imperial College, University of London, winning the University of London Granville Prize for Physics (top physics undergraduate degree in the University of London), before coming to Oxford to do a doctorate on pattern formation at the Centre for Mathematical Biology, Mathematical Institute. His thesis addressed a key problem with the classical Turing reaction–diffusion model for spatial pattern formation (Turing 1952), namely its inability to generate patterns in a robust and reliable fashion. Edmund proposed the idea that domain growth could play a prominent role in pattern selection. He was the first to rigorously formulate the Turing model on a growing domain and went on to show, using a combination of mathematical analysis and numerical simulation, that domain growth was able to allow the system to generate spatial pattern in a very robust way (see, for example, Crampin et al. 1999). He further generalised this work to look at different modes of growth, specifically spatially non-uniform growth (Crampin et al. 2002). Even though he then moved away from working on pattern formation, his formulation continues, to this day, to be used by research groups worldwide.

Edmund then decided to move into heart physiology, working with Denis Noble. He gained a (highly prestigious) Junior Research Fellowship at Brasenose College, Oxford, and an equally prestigious Wellcome Trust Research Fellowship. On completion of the latter, he moved to the Auckland Bioengineering Institute (ABI) at the University of Auckland in 2003. He led the Systems Biology research at the ABI, becoming Associate Director – Postgraduate in 2006. He also took up a lectureship in the Engineering Science Department in 2004 and was promoted to Associate Professor in 2010. His focus during his time in Auckland was on computational modelling at the level of ion channels, signal transduction pathways and gene regulation. Some of his most highly cited papers from this period are Crampin and Smith (2006), Cooling et al. (2007), Tran et al. (2009, 2010), Hurley et al. (2012) and Siekmann et al. (2012). Edmund’s wife Annalisa was also an ABI alumna, having undertaken her PhD at the ABI during this period. They subsequently had two children, Audrey and Peregrine, both born in Melbourne.

Edmund joined the University of Melbourne in 2013 as the Rowden White Chair of Systems Biology and Director of the Systems Biology Laboratory. This was a joint appointment between the School of Mathematics and Statistics and the Melbourne School of Engineering and set the scene for Edmund’s key role in bringing together researchers from across the mathematics, science and engineering fields to advance Systems Biology in all its aspects following the vision laid out by Kohl et al. (2010). In particular, he worked with engineers to develop thermodynamically based dynamic models (Gawthrop and Crampin 2014; Pan et al. 2021) and incorporate spatial effects (Rajagopal et al. 2015) and with mathematicians to model modal gating in ion channels (Siekmann et al. 2014) and cardiac calcium release (Hunt et al. 2020). This interdisciplinary research was only possible through Edmund’s encyclopaedic knowledge of disparate fields and his inspirational leadership.

Edmund was an innately highly talented individual who thought very deeply, not only about science, but about the world in general. He was also one of those rare scientists who was instantly engaging, in no small part because he was fully engaged with everybody he met, what they were doing and their aspirations. In doing so he made everybody feel their own work was more interesting and perhaps, even more significantly, that their dreams were both important and in reach. This interest in other people’s work, together with his enthusiasm, humour and profound insights, lit up any room he was in. Right from his time as a graduate student he fully engaged with those around him and he was an inspirational presence, helping fellow graduate students, giving novel ideas to new graduate students and filling the research group with positive energy. As he moved up the academic ladder he became a much sought after collaborator and supervisor. Professor Denis Noble at Oxford University, widely regarded as the father of Systems Biology, commented that Edmund was a leading light on what Systems Biology should be doing.

In addition to his significant academic achievements, Edmund was both an accomplished competitive cyclist and outdoor enthusiast. He was equally at home debating computational approaches as political ideologies, sometimes while writing papers with those fortunate enough to collaborate with him, sometimes eating dinner with those who were lucky to be his friends and sometimes while climbing mountains both literal and intellectual with those fortunate to keep up with him.

Edmund had been a member of the Editorial Board of the *Bulletin of Mathematical Biology* since 2010. He tragically passed away at the age of 47 on Saturday 15th May 2021 while on a bicycle ride.

**Portrait Caption:**

Portrait of Edmund Crampin, reproduced with the kind permission of the artist David Cobley (copyright).


